# Environmental Impact on Male (In)Fertility via Epigenetic Route

**DOI:** 10.3390/jcm9082520

**Published:** 2020-08-05

**Authors:** Matilde Cescon, Rosanna Chianese, Renata S. Tavares

**Affiliations:** 1 Department of Molecular Medicine, University of Padova, via U. Bassi 58/B, 35131 Padova, Italy; matilde.cescon@unipd.it; 2 Department of Experimental Medicine, University of Campania Luigi Vanvitelli, via Costantinopoli 16, 80138 Napoli, Italy; 3 Biology of Reproduction and Stem Cells Group, CNC—Center for Neuroscience and Cell Biology, University of Coimbra, 3004-504 Coimbra, Portugal; renata.tavares@cnc.uc.pt

**Keywords:** epigenetic signature of germ cells, sperm epigenome, environmental pollutant, diet impact on epigenetic route

## Abstract

In the last 40 years, male reproductive health—which is very sensitive to both environmental exposure and metabolic status—has deteriorated and the poor sperm quality observed has been suggested to affect offspring development and its health in adult life. In this scenario, evidence now suggests that epigenetics shapes endocrine functions, linking genetics and environment. During fertilization, spermatozoa share with the oocyte their epigenome, along with their haploid genome, in order to orchestrate embryo development. The epigenetic signature of spermatozoa is the result of a dynamic modulation of the epigenetic marks occurring, firstly, in the testis—during germ cell progression—then, along the epididymis, where spermatozoa still receive molecules, conveyed by epididymosomes. Paternal lifestyle, including nutrition and exposure to hazardous substances, alters the phenotype of the next generations, through the remodeling of a sperm epigenetic blueprint that dynamically reacts to a wide range of environmental and lifestyle stressors. With that in mind, this review will summarize and discuss insights into germline epigenetic plasticity caused by environmental stimuli and diet and how spermatozoa may be carriers of induced epimutations across generations through a mechanism known as paternal transgenerational epigenetic inheritance.

## 1. Introduction: An Overview of Epigenetic Mechanisms

Male infertility is one of the most common reproductive disorders strongly driven by environmental conditions before conception [1]. Although human health can be negatively impacted by environmental exposure at any time, there are sensitive windows of development during which every potential effect is amplified, namely prenatal, early childhood and puberty periods. For a long time, the burden of the offspring health has largely fallen on women, thinking at paternal preconception environment as insignificant. Recent studies point to paternal exposure as important for embryo development and offspring health outcomes [2].

Environmental exposures—including endocrine-disrupting chemicals and lifestyle exposures such as stress and diet—can alter the epigenetic marks in the germline, with spermatozoa (SPZ) as the final targets.

In comparison to the genome, cellular epigenetic landscape shows a high degree of plasticity, thus it is more influenced by the environment. The most commonly studied epigenetic mechanisms are the following: DNA methylation, histone modifications, chromatin folding and non-coding RNA expression.

In mammalian cells, the first DNA modification was described in 1948 by Rollin Hotchkiss as an “epicytosine”, anticipating the modern definition of epigenetics [3]. Such a modification was seen to correspond to a methylated cytosine. After that, in 1972, a new DNA base was identified and named 5-hydroxymethylcytosine (5 hmC) [4]. Although many researchers proposed DNA methylation as a mechanism regulating gene expression, it was not until the 1980s that several studies demonstrated that it was involved in gene regulation and cell differentiation [5]. CpG dinucleotides in non-coding regions, such as gene promoters, are the main targets of methylation; this chemical modification inversely correlates with the transcriptional activity of the locus. Conversely, actively transcribed genes are usually unmethylated, while a heavy methylation occurs in silenced genes [6]. However, controversial findings de-emphasize DNA methylation as the involved mechanism in gene repression with the evidence that the large majority of CpG-rich promoters are unmethylated in several cell types, regardless of state of expression [7].

Other corroborating studies demonstrate that DNA methylation is essential for silencing retroviral elements, regulating tissue-specific gene expression, genomic imprinting and X chromosome inactivation [8]. DNA methylation is carefully coordinated: the involved methylating enzymes are DNA methyltransferases (DNMTs) that transfer a methyl group from a S-adenyl methionine (SAM) to a cytosine residue to form 5-methylcytosine (5 mC). There are different forms of DNMTs: DNMT3a and DNMT3b establish a new methylation pattern to a previously unmodified DNA, which for this reason, are known as de novo DNMTs. On the other hand, DNMT1 functions during DNA replication to copy the DNA methylation pattern in a newly synthesized DNA strand [7]. Only the role of DNMT2 is still obscure. The reversibility of this enzymatic reaction has led to the discovery of an erasing DNA methylation mechanism, mainly controlled by Ten-Eleven Translocation (TET) enzymes that mediate active demethylation processes [9].

Chromatin organization is shaped by DNA packaged around histones [10]. These structural proteins can be post-translationally modified—as demonstrated in pioneer studies in the early 1960s—on their highly basic amino (N)-terminal tails, protruding from each nucleosome [11]. As a consequence, inter-nucleosomal interactions are affected and, thus, the overall chromatin folding. Histone post-translational modifications (HPTMs) are known to alter gene expression and to be finely regulated by key enzymes [12]. The most extensively studied HPTMs include methylation, acetylation, phosphorylation and ubiquitination. Interestingly, each modification—taking place on specific amino acid residues—changes its significance in terms of gene expression; for example, histone H3 methylated at lysine 9 is a hallmark of transcriptionally inactive DNA, whereas histone H3 methylated at lysine 4 is associated with transcriptionally active genes [13]. Therefore, chromatin structure shows a high degree of plasticity, tightly controlled by a large number of possible HPTMs. Interestingly, a crosstalk between different modifications exists, adding an extra level of complexity in gene expression regulation [14].

It is now well-established that only a small subset of the human genome (1–2%) includes protein-coding sequences, while the remaining is constituted by regulatory DNA sequences transcribed into an unexpected variety of non-coding RNA (ncRNA) molecules, introns, transposons, repeats and other sequences whose function is yet far from being completely determined [15].

Recent advances in molecular biology, through the development of next generation sequencing technologies and supported by bioinformatic analyses, have shown several classes of ncRNAs involved in transcriptional regulation, in several physiological and pathological processes as well as in different biological contexts. They are divided in long (>200 nucleotides) and small (<200 nucleotides) ncRNAs [16]. In the context of small ncRNAs, there are two additional classes: small housekeeping (tRNAs, small nucleolar and nuclear RNAs) and small regulatory ncRNAs (PIWI-interacting RNAs, microRNAs and circular RNAs, namely piRNAs, miRNAs and circRNAs, respectively). Interestingly, most of them constitute regulatory networks, where the abundance of one RNA affects the abundance and the activity of others. Considering the potential implication of each RNA component in multiple signaling pathways, it is clear that RNA network activation may have amplified effects in cells [17]. One of the most acclaimed RNA network type is composed by circRNAs-miRNAs-mRNAs, also known as competitive endogenous RNA (ceRNA) network (ceRNET). In addition, a crosstalk between DNA methylation and histone modifications has also been suggested [18]; for example, DNA-methylated promoters recruit protein complex containing histone methyltransferases in order to induce chromatin structural changes through histone modifications [19]. Moreover, various ncRNAs are also closely associated with other epigenetic marks, in turn forming an “epigenetic network” [20].

Genome accessibility is, therefore, under the control of the cell epigenome. Such control is, nevertheless, more complicated than first thought since it results not only from multiple epigenetic mechanisms, but also by their interactions.

## 2. Epigenetic Signature of Male Germline

Sperm is more than just a vehicle of a haploid genome. It is equipped with a very peculiar epigenetic landscape, susceptible to environmental exposures and modulating the developmental programming of the offspring by the so-called intergenerational (if information is passed between two generations) or transgenerational (if information is passed across multiple generations, usually three or more) epigenetic inheritance [21]. During male germline development, several cellular changes take place. Germ cells undergo impressive morphological and physiological modifications in order to become high specialized cells—the SPZ. Research is nowadays addressed to counteract the dogma that SPZ are only responsible for the safe transmission of the paternal DNA. Contrasting evidence is increasing and suggests that SPZ have their own epigenome, dynamically established during spermatogenesis [22]. As spermatogenesis progresses, paternal DNA methylation undergoes waves of demethylation and de novo methylation; DNA packaging is orchestrated by protamines, even if a part of paternal histones is still retained, and a wide spectrum of RNAs has been detected in SPZ, although transcriptionally quiescent. Even more interesting, epigenetic changes in sperm are strongly correlated to sperm quality and embryo development and create a cellular signature inherited by the offspring through an intergenerational transmission [23,24].

### 2.1. Epigenetic Mechanisms during Germ Cell Progression

DNA methylation, histone modifications and chromatin reorganization, noncoding RNA regulation are the major epigenetic mechanisms that there will be described during the progression of the spermatogenesis.

#### 2.1.1. DNA Methylation

There are two major reprogramming events that involve genome-wide erasure and re-establishment of DNA methylation patterns during mammalian development. The first one occurs in primordial germ cells (PGCs) during their proliferation and migration to colonize the genital ridge, and the second after fertilization in the pre-implantation embryo [25]. In what concerns the first, the PGC genome is globally demethylated resulting in negligible levels of DNA methylation [26]. From here, the process of sexual dimorphic development starts and involves extensive DNA remethylation. In males, some pro-spermatogonia within the basement membrane of the seminiferous tubule resume mitotic divisions to form primary spermatocytes. Secondary spermatocytes are then formed by meiotic division, dividing once again to give rise to haploid spermatids. The remaining pro-spermatogonia continue to divide mitotically and produce spermatogonial stem cells (SSCs) to ensure spermatogonia pool. Finally, in the last phase of spermatogenesis—a series of morphological rearrangements occur involving the loss of most cytoplasm and some organelles, microtubule growth, tail and acrosome formation, and dramatic chromatin packaging [27]. These periods of reprogramming occur during critical developmental timeframes and represent windows of susceptibility for epigenetic errors to occur, possibly affecting fertility and embryonic competence [28].

As previously described, three DNMT families control the methylation in mammalian cells. DNMT3l, an isoform of DNMT3a and DNMT3b but with no enzymatic activity, has also been found essential for spermatogenesis as its absence avoids spermatogonial progeny from undergoing differentiation and meiosis. In fact, just during meiosis, DNMT3a, DNMT3b, and cofactor DNMT3l activity regulates the levels of de novo DNA methylation, completing this process after birth at the stage of pachytene spermatocyte [29]. Subsequently, the methylation profile is maintained by DNMT1 activity. In the mammalian genome an important phenomenon termed “genomic imprinting” regulates the expression of some genes, leading to monoallelic parental-dependent expression of imprinted genes [30]. After DNA demethylation during PGC development, sperm and oocyte-specific methylation marks need to be re-established to prevent imprinting disorders associated with altered dosage of imprinted gene expression in the progeny [31,32,33]; this process occurs at their imprinting control regions (ICRs). For instance, under normal circumstances, *H19*-*Igf2* and *Meg3* imprinted genes appear hypermethylated in sperm, as well as the repeat element intracisternal A particle IAP [34] and hypomethylated in oocytes. In the opposite direction, *Lit1*-, *Snrpn*- and *Mest*-imprinted genes appear hypomethylated in SPZ and hypermethylated in oocytes. However, aberrant methylation patterns at these genes might occur, and this has been associated with male sub/infertility [35].

DNA methylation has been the most comprehensively studied epigenetic mark so far, however HPTMs and the role of ncRNA molecules are recently gaining considerable attention.

#### 2.1.2. Chromatin Remodeling

**Figure 1:**
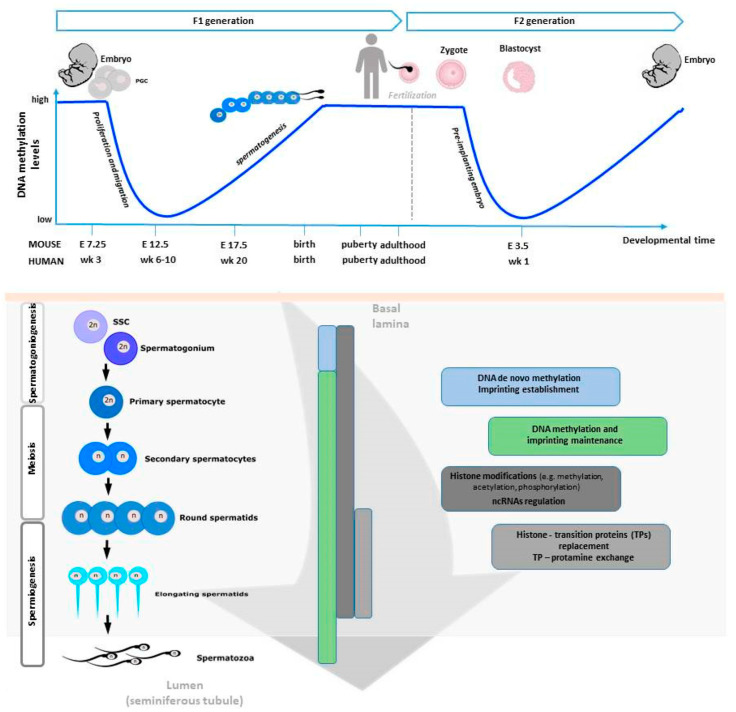
Overview of the epigenetic regulation of spermatogenesis. Starting from primordial germ cells (PGCs), germ cell progression occurs during mitosis, meiosis and spermiogenesis. In each step, important epigenetic changes take place, most of them are retained in the spermatozoa (SPZ) before they start to travel along the epididymis.

As explained later, during spermiogenesis, chromatin will undergo a strong remodeling consisting in the incorporation of testis-specific histone variants, replacement of histones with transition proteins, protamine incorporation and HPTMs [36]. Interestingly, a methylation pattern for histone H3 has been described during meiosis [37], whereas during spermiogenesis the main HPTM is the acetylation that plays a crucial role for correct histone to protamine transition and allows nucleosome disassembly in elongating spermatids [38]. In humans, two isoforms of protamines exist —P1 and P2. Previous studies have shown that in a balanced spermatogenic process the mean P1/P2 ratio is approximately 1.0 in human sperm [39,40,41]. Nevertheless, infertile men exhibit modified P1/P2 ratios and/or undetectable P2 by contrast with the fertile ones in which such anomalies are uncommon [41,42,43,44,45,46]. Additionally, altered spermatogenesis and male infertility have been found in transgenic mice with protamine haploinsufficiency [47]. Figure 1 is a schematic view of the epigenetic regulation occurring during spermatogenesis.

#### 2.1.3. NcRNAs

In the scenario of ncRNAs, miRNAs (19–25 bp in length)—acting as mRNA translation suppressors or mRNA breakdown inducers in mammalian cells—are undoubtedly important for spermatogenesis given that a global loss of miRNAs through the generation of *Dicer* knockout mice have shown deleterious effects on male fertility [48]. In animals, miRNA genes are typically transcribed by the RNA polimerase II and require Dicer to form double-stranded mature miRNA. Then, usually one strand of the miRNA is preferentially loaded into the effector miRNA-induced silencing complex (miRISC). The miRISC complex, that comprises Argonaute (AGO) proteins, mediates the post-translational regulation of mRNA targets of the loaded miRNA [49]. In sharp contrast, piRNAs (mostly 24–34 bp) interact with the PIWI instead of AGO proteins and have been shown to function independently of Dicer. Nevertheless, just like miRNAs, piRNAs are important regulators of male germ cell differentiation [50]. Supporting this evidence are, once again, studies involving knockout animal models. These show that PIWI proteins (MILI, MIWI2 and MIWI) are essential for the successful completion of spermatogenesis [51]. Indeed, ncRNAs are able to bind to the evolutionarily conserved proteins of the PIWI/AGO family. While PIWI proteins bind to piRNAs, as previously mentioned, and are highly enriched in the germline, AGO proteins can be present in both somatic and germ cell binding, not only to miRNAs, but also to short interfering RNAs [50]. Compelling data have determined that abnormal miRNA regulation is associated with male infertility. By analyzing testicular miRNA profiles from both normal healthy males and patients presenting some kind of reproductive issue (e.g., asthenozoospermia, Sertoli cell only syndrome, mixed atrophy, and germ cell arrest) a large set of dysregulated miRNAs has been observed [52,53,54]. Curiously, many single-nucleotide polymorphisms (SNPs) have been detected in miRNA-binding sites of candidate genes crucial for male fertility, which in turn may potentially affect the expression of these genes and enhance the risk of male infertility [55]. Coupled to this, SNPs in Dicer and Drosha, an RNase III endonuclease that cleaves specific stem loop structures of monocistronic primary transcripts (pri-miRNA) to form isolated hairpin loops (pre-miRNA), have also been associated with sperm quality, further strengthening the crucial role of miRNAs in male fertility [56]. Moreover, several SNPs in human *PIWI* genes have also been found associated with the risk of spermatogenic failure or increased risk of oligozoospermia [57]. In accordance, other studies found that allele-specific DNA methylation differences in PIWIL1 and PIWIL2 are related to altered spermatogenesis and male infertility [58]. Altogether this evidence contributes to the knowledge that genetic variations in piRNAs may also affect human male fertility.

Each stage of germ cell progression has been systematically analyzed in terms of circRNA content. Interestingly, 15,101 circRNAs have been detected in mouse spermatogenic cells with a dynamic pattern starting from spermatogonial germ cells to spermatids, with the higher number especially in round spermatids, thus to hypothesize an important control of circRNAs in their maturation [59]. A similar profile has also been described in rats demonstrating that (i) circRNAs are evolutionarily more conserved than linear mRNAs; (ii) circRNAs have higher tissue specificity than mRNAs; (iii) in testis, circRNA expression dynamically changes depending on the age: it increases with sexual maturity and decreases with aging [60]. Human testes are also enriched in circRNA content and testis-derived circRNAs stably exist in seminal plasma, probably in the form of protein complexes, thus strongly suggesting their application as novel non-invasive biomarkers for male fertility [61].

Long ncRNAs (lncRNAs) are important regulatory factors lacking functional Open Reading Frames (ORFs) and localized in both the nucleus and cytoplasm [62]. Yet, despite the evidence that lncRNAs are important players in diverse cellular processes, the in vivo functional characterization of lncRNA are still experiencing several difficulties [63]. At this point, in the testis, it is known that some lncRNAs play a critical role in SSC self-renewal, in both humans and mice [64,65], but much is still undisclosed.

### 2.2. Epigenetic Mechanisms in Mature SPZ

Mammalian SPZ are extraordinarily specialized cells with peculiar features: a markedly condensed nucleus accompanied by the secretory vesicle acrosome, little cytoplasm and a long flagellum. These cells complete in testis an impressive series of cellular, molecular and morphological changes during spermiogenesis in order to become streamlined cells able to fertilize oocytes [66]. Outside the testis, SPZ, still immature, acquire novel competences travelling along the epididymis where they encounter a different extracellular milieu modulating their biochemical composition and functionality [67].

The most classical idea about the biological function of SPZ has been to transmit the paternal genetic information during fertilization. Several reports have nowadays demonstrated that SPZ are invested with an epigenetic fingerprint that is difficult to think do not get involved in early embryonic development; rather, it has the potential to be transferred intergenerationally or transgenerationally [28]. If this is true for several species, mammals included, the transmission of the epigenetic information across generations in humans should take into account genetic, ecological and cultural inheritance as well, beyond the consolidated molecular mechanisms, thus making harder any consideration in this regard [68].

Chromatin in sperm is structurally distinct from that of somatic cells. In fact, during spermiogenesis DNA-binding histones are progressively replaced by transition proteins followed by protamines, so that chromatin reaches its six-fold compaction, in order to confer a higher protection to paternal genome from external insults and nuclease activity [69,70]. Such a substitution leads to a chromatin structure known as “doughnut loop” or “toroid”, that allows SPZ nucleus to acquire a hydrodynamic shape, favorable to their journey along male and female tracts [71]. In order for this dynamic process to occur, histone modifying enzymes determine combinations of HPTMs, creating specific signals that define the so-called “histone code”. This, in turn, will induce specific changes in chromatin structure and function. Essentially, this code proposes that combinations of histone alterations could define specific signals for chromatin domains. This is further supported by the discovery of chromatin-interacting modules specifically recognizing methylated or acetylated histone lysines [72]. In fact, all core histones have several sites for potential posttranslational covalent modifications at specific residues in their N-termini, including acetylation, methylation and phosphorylation [73]. While histone acetylation is generally associated with transcriptionally active regions of the genome, histone methylation can be associated with active or repressed regions depending on the residue being methylated. Histone acetylation is carried out by histone acetyltransferases (HATs) and the reverse process, termed deacetylation, is performed by histone deacetylases (HDACs). In the same way, the histone methylation is carried out by histone methyltransferases and the reverse by histone demethylases. Whereas a lysine residue can have only one acetyl group, a lysine residue can be modified with mono-, di-or (tri)-methylation [74]. A growing number of enzymes have been held responsible for histone modifications [73].

Histone to protamine transition is, then, favored by a massive acetylation of the histone H4 and is accompanied by the di-methylation of histone H3 and chromatin opening; however, during this repackaging, a small percentage (5–10%) of DNA is still organized in nucleosomes thanks to a residual presence of histones [75]. Prior to histone eviction, in spermatids, several histone variants are also incorporated: H2AX and its phosphorylated form, γ-H2AX, associated with the sex body, closely involved in the inactivation of the sex chromosomes during spermiogenesis [76]; TH2B associated with telomeres [77]; CENP-A, a variant of histone H3, that decorates centromeres [78]. Interestingly, residual histones undergo chemical post-translational modifications that drastically alter their DNA binding abilities [74,79,80]. In transcriptionally inactive cells as SPZ, high levels of acetylated histones—usually a characteristic feature of transcriptional activity [81]—have no clear functions, unless they could represent a sperm-specific epigenetic mark controlling gene expression in the zygote. Accordingly, all the other HPTMs in sperm chromatin act as a “tag”, a sort of “stable memory” of the paternal epigenetic information inherited by the offspring; Chioccarelli et al. deeply discuss HPTMs in SPZ as well as their implication in sperm quality and fertility [74]. Sperm quality also depends on the protamine P1 and P2 ratio as well as on the correlation between specific histone profiles and protamine levels, as in the case of P2 and H3K9me2 (di-methylation of histone H3 on lysine 9) [82,83]. Interestingly, persisting histones are not randomly located into the genome, but rather maintained during fertilization and enriched at promoters of genes involved in early embryonic development [84]. Remarkably, an excessive histone retention is correlated to infertility [85]. In mouse sperm, histone retention mainly occurs at regions of high CpG density and low DNA methylation [75]. Subsequent to fertilization, sperm chromatin is quickly remodeled due to the replacement of protamines with maternally derived histones.

In the sperm nucleus, Scaffold/Matrix Attachment Regions (MARs) are organized neither in toroidal structures nor in nucleosomes, creating another level of chromatin folding [86]. These regions constitute attachment points of loop domains of DNA, a structural support to the chromatin that in the sperm nucleus is an additional mark inherited by the embryo and required for proper embryogenesis [87].

**Figure 2:**
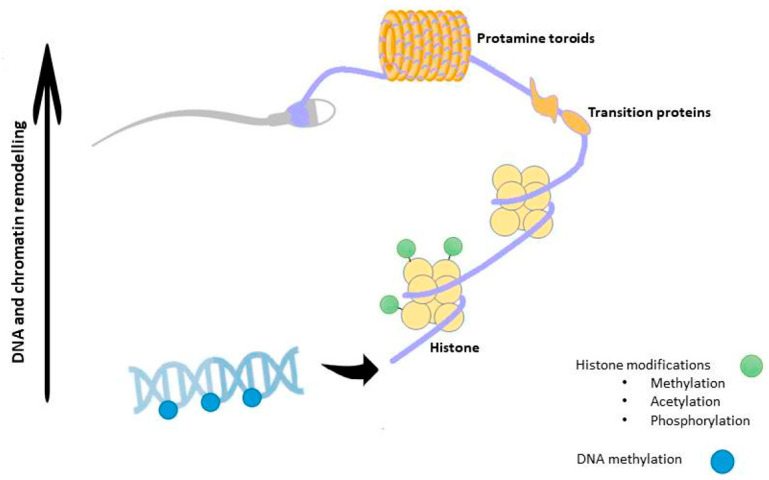
A schematic view of chromatin remodeling in SPZ. During spermiogenesis, cells suffer chromatin conformational changes regulated by epigenetic marks, ultimately resulting in an extremely condensed chromatin that protects SPZ from external assault. In this last step of spermatogenesis DNA is methylated, histones suffer posttranslational modifications and are replaced by transition proteins (TPs), an intermediary state of packaging that soon disappears. TPs are then exchanged with more basic proteins, the protamines. DNA strands are tightly wrapped to protamines, forming supercoiled doughnut-like structures named toroids, the basic packaging units of sperm chromatin. Of note, a small subset of histones escapes this fate and is not replaced.

At the end of spermatogenesis, SPZ are highly methylated (approximately 90% of CpG islands are methylated in mouse and 70% in fully mature human SPZ) in comparison with somatic cells and oocytes that, on the contrary, have only 40% of methylated CpGs [88]. Interestingly, the global methylation in sperm is highly gene specific, thus playing a role in directing transcription in the early embryo, abundant at intergenic regions and uncommon at gene promoters [89,90]. Additionally, sperm-specific hypomethylated regions are in genes related to germ cell development [91]. However, DNA methylation pattern is highly dynamic. In fact, quickly after fertilization DNA methylation is globally erased and at a much faster rate than the maternal one [90]. At the level of imprinted genes, DNA methylation escapes the genome-wide reprogramming that occurs after fertilization [92]. Several studies have correlated defects in sperm DNA methylation with failures of spermatogenesis, reduced sperm quality, count and motility [93]. Infertile men also contain a higher rate of 5 hmC than fertile ones, with an increased DNA fragmentation rate, as a consequence [94]. Several reports have suggested that the assisted reproductive technology (ART) may encourage the birth of children with rare epigenetic syndromes, mainly owing to use of sperm with imprinting disorders, thus confirming that this procedure can lead to the formation of primary epimutations, via SPZ, as will be discussed below [95,96]. Such an evidence emphasizes the importance of a deep evaluation of sperm quality, at both genetic and epigenetic levels, while performing these techniques. Aberrant DNA methylation also correlates with pregnancy failure [97]. Chromatin remodeling influenced by DNA methylation and HPTMs is outlined in Figure 2.

Another category of epigenetic regulators of gene expression is the family of ncRNAs. A recent review deeply summarizes the vast repertoire of RNA molecules enriching SPZ [74]. Their existence has been highly debated since the transcriptionally dormant state of the sperm nucleus and the ejection of most of the cytoplasm and RNA content at the end of spermatogenesis. Despite that, SPZ retain a complex entourage of mRNAs that, interestingly, persist in a translationally silent cell, suggesting controversy in these discoveries [98]. About the significance of sperm mRNAs, opinions are rather divided: although many andrologists remain unconvinced by the phenomenon attributing sperm transcripts to the presence of residual cytoplasmic droplets that escape absorption by Sertoli cells, many other strongly believe that sperm mRNAs may be involved in sperm physiology, regulating both motility and capacitation [99], selective chromatin repackaging or genomic imprinting [98], in addition to be delivered to the oocyte during fertilization, in order to control early embryonic development [100]. Interestingly, a differential expression of sperm mRNAs between fertile and infertile individuals should illuminate new genes and gene pathways underpinning male infertility, making these RNAs potential biomarkers for fertility evaluation [101,102].

In addition to mRNA, in SPZ there are numerous small regulatory ncRNAs, mainly miRNAs and piRNAs. However, recent investigation has pointed to other ncRNA classes that populate SPZ: tRNA-derived small RNAs (tsRNAs) [103], ribosomal RNA (rRNA)-derived small RNAs (rsRNAs) [104] and circRNAs [105,106]. Most of SPZ-borne ncRNAs are synthesized during spermatogenesis, but the high degree of plasticity displayed by sperm ncRNA profile suggests an epididymal contribution—via epididymosomes—during SPZ maturation [107]. In the scenario of ncRNAs, circRNAs pave attention. Thought to be junk by-products in gene transcription, they actually represent the largest RNA family in human transcriptome [108], with a regulatory role trapped in a complicated network involving mRNAs, miRNAs and proteins. CircRNAs have been discovered in human embryos [109,110] and are potentially involved in chromosome organization, cell cycle regulation and DNA repair. First considered a maternal contribution to the embryonic inheritance, they have been now characterized in mouse and in human SPZ, collected from both normozoospermic and asthenozoospermic subjects [105,106,111]. Indeed, due to the differential pattern of expression observed, circRNAs have been suggested as a possible sperm quality markers [105,111]. Interestingly, paternal circRNAs may be transferred from SPZ into the oocyte during fertilization as in the case of SPZ-derived circNAPEPLDiso1, a circRNA of NAPEPLD, the enzyme involved in the biosynthesis of the endocannabinoid anandamide (AEA) [112], which has key roles in reproductive functions [113,114,115,116,117,118]. In comparison to unfertilized murine oocytes, the expression of circNAPEPLDiso1 significantly increases in fertilized oocytes, suggesting a paternal contribution [106]. Given their ability to physically interact with miRNAs primarily involved in the control of cell cycle, it is plausible its contribution just in the first stages of embryo development [106].

In addition to sperm-borne RNA repertoire, mammalian SPZ also deliver to the offspring a rich combination of nuclear proteins [118,119]. Besides histones and protamines, SPZ possess an impressive proteomic landscape which is postulated to contribute to the epigenetic landscape of these cells [109]. The advancement of proteomic techniques based on mass spectrometry has surely favored the discovery of hundred proteins in isolated human sperm head fractions [118,120]. A Gene Ontology analysis indicates that more than 50% of them have potential epigenetic activity because they are involved in chromatin organization, protein–DNA complex assembly, gene expression and histone modifications [118]. Such an analysis also shows a significant enrichment in biological processes related to histone acetylation and gene expression, that is amazing since SPZ are historically considered transcriptionally and translationally inert cells [121]. TATA-box binding proteins, zinc fingers and bromodomain-containing proteins, elongated factors and RNA polymerase II in mature SPZ may be considered simply leftovers from past events of spermatogenesis or could have a role in the regulation of transcription after fertilization and during early embryo development. This aspect is still more intriguing in the light of the large set of coding and ncRNAs delivered by the SPZ into the oocyte, originally thought to be an incomplete expulsion of cytoplasmic elements during nuclear condensation [122,123]. Several other DNA-related proteins have also been found, such as enzymes involved in DNA methylation, replication and repair [118]. During their journey, SPZ are immersed in the seminal fluid that does not function only as a transport medium since it contains several sperm-derived factors, embedded in exosomes, proposed to influence fertilization, embryo development and offspring health [124].

### 2.3. Alterations of Epigenetic Mechanisms Cause the Spermatogenic Failure

Germ cell epigenetic signature is surely a quality marker to check. Recent studies have focused on discovering how alterations of epigenetic mechanisms may impact sperm quality causing infertility. Evidence from both animal models and humans exists about a correlation between sperm DNA methylation and male infertility [125]. Disturbed spermatogenesis has been, indeed, associated with incorrect imprinting as a result of DNA methylation dysregulation [126,127]. Low methylation of *H19* and high methylation of *Mest*-imprinted genes have been observed in SPZ of oligozoospermic patients [128]. Similarly, DNA methylation-mediated wide genomic imprinting is frequently altered in men with oligoasthenoteratozoospermia and oligozoospermia [129,130]. A molecular analysis of genome wide alterations in sperm DNA methylation has been used to identify male idiopathic infertility, being also responsive to follicle stimulating hormone (FSH) therapeutic approach [131].

Sperm chromatin remodeling is also altered in subfertile/infertile patients. Protamine content plays a critical role in chromatin condensation. A species-specific P1/P2 ratio exists in both mice and humans; its alteration is associated with subfertility [41]. Interestingly, *Prm-2* deficient mice not only display impaired histone to protamine exchange and disturbed DNA-hypercondensation, but also severe membrane defects resulting in sperm immotility and irregular sperm head morphology [132]. The abnormal histone retention is one of the most affected parameters linked to male infertility: it implies an alteration in protamine content, indicative of poor semen quality, and leads to atypical rearrangements in chromatin organization of developmental loci and genes, with a strong impact on normal embryo development [41,133]. Remarkably, the altered histone content in infertile patients has also been demonstrated by a proteomic approach [134]. In particular, an unusually high abundance of histone variants has been identified as an epigenetic error contributing to failed embryo development [134]. Beyond histone variants, the complex picture of histone modifications has also been described as an important quality marker of sperm, as recently reviewed [74,135].

As laid out above, a set of functional RNAs has been characterized in mature SPZ with the evidence that they are delivered into oocytes, contributing to early embryo development and, thus, influencing the phenotypic traits of the offspring. Poor semen quality has been associated with higher sperm RNA content [136]. Accordingly, tsRNAs, rsRNAs, and miRNAs show a differential expression, linked to sperm quality, according to embryo quality, regardless traditional semen-parameter assessment [137]. This result is perfectly in line with evidence about circRNA content, much richer in asthenozoospermic patients, in comparison to normozoospermic individuals [105,111]. Unlike what has been thought for decades, sperm RNAs provide crucial information on sperm competence, embryo development and infertility etiology [138,139].

## 3. Epigenetic Mechanisms Are Sensitive to Environment and Lifestyle

Global society must face the impact of pollutants and the change in its own lifestyle as two important threats to its health.

Environmental contaminants, usually endocrine disrupting chemicals (EDCs), are able to mimic or antagonize endogenous hormones, affecting neurological, cardiovascular, immune, reproductive and developmental health of both humans and animal populations that are continuously exposed to ubiquitous synthetic and natural-occurring EDCs through diet, dermal contact and/or inhalation [140,141].

Furthermore, modern society is shifting its own eating habits in terms of a higher calorie intake and the ingestion of highly processed foods and animal products. These dietary shifts are often the cause of several diet-related diseases [142].

Maternal and paternal exposure to environmental contaminants has deleterious effects on the offspring health in subsequent generations. Although EDC effects are likely induced via multiple genomic-based pathways, their non-genomic effects are also relevant. Since SPZ are transcriptionally and translationally inactive cells, they are a good model to address these non-genomic effects [141].

Actually, reproductive health is in the crosshair of EDCs; EDC-mediated adverse effects on fertility are more prominent in obese individuals, suggesting a dual suppressive action of diet and environment on SPZ physiology.

What is intriguing is that epigenetic routes—starting from spermatogenesis to mature SPZ in epididymal transit—are especially targeted [143]. Nowadays, the expression “Environmentally epigenetics” is used to refer how environmental exposures—which can be pollutants and lifestyle factors—consequently shape epigenetic changes and gene expression [144].

### 3.1. Environment Induced Epigenetic Modifications Targeting Spermatogenesis

Compelling data have nowadays shown that many factors can alter the epigenetic programming of male germ cells, thus posing a great threat to mammalian development. In this regard, EDCs have a prominent role. These compounds possess antiandrogenic or (anti)estrogenic-like activities and affect the action of endogenous hormones, leading to hormonal imbalance through diverse mechanisms. Furthermore, as a highly heterogeneous group of compounds that range from naturally produced—including mycotoxins and phytoestrogens—to synthetic substances—such as pesticides, polychlorinated dibenzo-p-dioxins, polychlorinated biphenyls (PCBs), polycyclic aromatic hydrocarbons (PAHs), bisphenol A (BPA), phthalates and heavy metals, they are ubiquitously distributed in the environment, being present in air, soil and contaminated food and water [141].

With the purpose of avoiding, abolishing, or controlling any pest and obtaining higher crop yields, mankind has developed a wide range of pesticides. However, these compounds have been shown to affect male reproductive function in humans and animal models, among other endocrine systems. For instance, the herbicide atrazine is the most common contaminant found in underground waters in the USA [145] and it is metabolized in mammalian organisms by Glutathione S-transferases and cytochrome P450 systems [146,147]. In mammals, atrazine may affect several tissues, ranging from testes [148,149,150] and ovaries [151,152,153] to brain [154] and liver [155,156,157], and the presence of its metabolites has been already associated with low birthweight in humans [158]. Atrazine affects meiosis, spermiogenesis and reduces sperm output in mice following in utero exposure [159] and this could actually be related with both the observed dramatic deregulation of RNA transcription in the testis and the global decrease of H3K4me3. Importantly, these changes could have started in the undifferentiated spermatogonia and led to the decreased phenotype observed [159]. On the other hand, p,p’-Dichlorodiphenoxydichloroethylene (p,p’-DDE)—the principal and most stable metabolite of the organochlorine pesticide DDT—is an extremely persistent compound that is de novo introduced in the environment still via DDT use in some developing countries [141]. As a known androgen receptor antagonist, p,p’-DDE may alter anogenital distance and nipple retention in male offspring after gestational exposure [160]. Furthermore, still during pregnancy p,p’-DDE impairs testis histology and male fertility, inducing *Igf2* hypomethylation in sperm [161]. More recently, besides *Igf2* hypomethylation, p,p’-DDE was shown to directly promote hypomethylation of the *H19*-imprinted gene, but with no differences in what concerns *Snrpn* and *Peg3* genes. p,p’-DDE has also been implicated in spermatogonia impairment in prepubertal and pubertal rats [162,163].

The fungicide vinclozolin has long been associated with aberrant DNA methylation patterns that will be carried by SPZ to the future generations, as discussed later in this review. Nevertheless, Sertoli cells are also affected [164]. Indeed, over 400 genes are differentially expressed, and a number of specific cellular pathways are found altered. Furthermore, the epigenome of these cells is also modified with differential DNA methylation patterns in more than 100 promoter regions. One of the processes affected is the production of pyruvate/lactate that is used as the primary energy metabolite by the germ cells that, by being trapped within the blood–testis barrier, cannot acquire glucose [165,166]. Consequently, this will have implications in germ cell viability and, thus, spermatogenesis development [164]. Lactational exposure to the PCB Arochlor 1254 has also been found to affect Sertoli cells by decreasing the expression of both follicle-stimulating hormone receptor (FshR) and androgen receptor (AR); nevertheless, the level of transcription factors regulating *FshR* and *AR* gene expression, and DNA methylation in the promoter of these genes in both prepuberal and puberal rats were not known until recently [167]. Priya and colleagues found that the transcription factors that regulate the *FshR* and *AR* genes are reduced in both PCB-exposed groups and methylation has been detected in the promoter of *FshR*, *AR* and steroidogenic factor 1 (*Sf1*). Furthermore, the protein levels of the enzymes DNMT1, DNMT3ab, DNMT3l, and Hdac1 are increased in the treated groups, consequently leading to the transcriptional gene repression observed in the Sertoli cells [167].

Carbendazim and chlorothalonil are other fungicides that may compromise male fertility but whose mechanisms are not fully understood. Reports from last year and early 2020 have shown that low doses of each compound can disrupt the spermatogenesis of pubertal mice via estrogen receptor (ER) signaling modulation which potentially leads to the disturbance of the global DNA methylation and histone methylation observed in mice [168,169]. Synergistic effects have also been reported to occur in pubertal mice orally exposed to low doses of both compounds [170].

BPA, perhaps the most studied compound in this context [171,172], can be found in contaminated food and water and released from industrial products by various physical or chemical processes, including heating, aging, light exposure and/or contact with either acidic or alkaline compounds [173]. Apart from being detected in many biological fluids, more than 90% of human urine samples contain traceable amounts of BPA [174], indicating the importance of analyzing BPA-induced health effects.

Exposure to BPA during critical windows of development, i.e., for the definition of epigenetic marks, may impact the epigenetic signature and consequently the health of the exposed organism and/or their offspring. Indeed, similarly to the pesticides carbendazim and chlorothalonil, BPA is suggested to alter reproductive function through ER modulation. Neonatal exposure to BPA has been shown to alter methylation of the ER alpha promoter and enhance the expression of the enzymes DNMT3a and DNMT3b at both transcript and protein levels in adult rat testis, suggesting aberrant DNA methylation at several gene loci [175]. Furthermore, to test if BPA-induced reproductive impairment could be associated with the altered expression of steroid receptors and corregulators in testes, male rats have been perinatally exposed to BPA [176]. A significant reduction in the expression of the steroid receptor coactivator-1 (Src-1) and nuclear coreceptor (NCoR) with a parallel increase in the expression of p/CIP (p300/CBP/cointegrator-associated protein) and G-receptor integrating protein-1 (Grip-1) has been observed in testes [177].

In order to unveil the molecular mechanisms underlying BPA-induced spermatogenesis dysfunction and reduced sperm quality, studies solely targeting spermatogonia and spermatocytes have been carried out. In mouse spermatocyte-derived GC-2 cells, BPA has been found to inhibit DNA replication, triggering apoptosis and oxidative stress. Furthermore, a global increase of both genome-wide and locus-specific methylation in these spermatocytes has been reported [177]. Additionally, exposure to 10 µg/mL BPA, but not to lower concentrations, significantly inhibits cell growth and promotes a reduction in global DNA methylation levels in the GC-1 spermatogonial cell line. The protein and mRNA expression levels of DNMT1 decrease as well as the global levels of H3K27me3. In contrast, increased phosphorylation of p38 and decreased phosphorylation of extracellular signal-regulated kinases 1/2 have been observed, demonstrating a modulation of the MAPK-signaling pathways [178].

Recently, sirtuin 1 (Sirt1) has been implicated in BPA-induced effects in male reproductive tract. The rationale is simple: Sirt1 has an essential role in germ cells differentiation and fertility, thus an increase in the expression of this well-known class III-HDAC can possibly mediate the decreased acetylation levels of H3 and H4 histones observed in chronically BPA-treated rats [179]. Indeed, chronic exposure to a low BPA concentration can impact the first round of spermatogenesis through Sirt1 modulation [180]. First via placenta and then through lactation and drinking water, BPA is able to impair the blood-testis-barrier, induce the production of reactive oxygen species (ROS) and DNA damage and diminish Sirt1 expression. Analysis of Sirt1 downstream molecular pathways shows increased acetyl-p53Lys370, γH2AX foci, decreased oxidative stress defenses and higher apoptotic rate in rat testis, with partial rescue at sex maturation. Importantly, the concentration used herein is lower or within the reference limit for humans and therefore, currently considered “safe” by the European Food Safety Agency (ESFA) and the U.S. Environmental Protection Agency (EPA) [180]. Nevertheless, it retrieves worrisome results.

Finally, few data are available concerning the effects of BPA on miRNA/tRF expression in testis. Yet, BPA exposure for 24 h induces a significant downregulation of miRNA expression patterns in the TM4 Sertoli cell line during the course of treatment [181].

Already in 2000, Quintanilla-Veja and co-workers suggest that heavy metals, particularly lead, could compete with or replace the natural component zinc in human P2 in vivo, resulting in a dose-dependent decrease in the extent of P2-DNA binding and consequent alteration in chromatin condensation [182]. Since then, limited information became available on the capacity of heavy metals to induce epigenetic changes. Indeed, only very recently cadmium, a known male reproductive toxicant, has shown to induce massive and aberrant lncRNA and mRNA expression profiles in testes and SPZ of C57BL/6J mice, concomitant with the observation of decreased testicular sperm production, motility and normal morphology [183]. 139 and 174 lncRNAs are up- and downregulated, respectively, in the testes of the exposed mice and, in contrast, 214 mRNAs have been found up- and 226 downregulated in the same tissue. As for sperm cells, higher numbers of lncRNAs are up- and downregulated and only 272 and 111 mRNAs are up- and downregulated, respectively. Gene ontology and pathway analyses determine that the targets of these differentially expressed lncRNAs and mRNAs are closely related to processes involved in spermatogenesis. Furthermore, Gao and colleagues also found that several newly identified lncRNAs show inducible expression, making them good candidate markers for analyzing cadmium-provoked male reproductive toxicity [183]. Additionally, the toxic effect of hexavalent chromium (Cr(VI)) on mouse spermatogonial stem cells has also been recently addressed. A global increase in both H3K9me3 and H3K27me3 has been observed as well as activation of the apoptotic signaling pathway. Nevertheless, in vitro pretreatment with melatonin attenuates (Cr(VI)) induced increase of histone methyltransferase ESET abundance, besides diminishing apoptosis and the global increase of H3K9me3. Furthermore, exogenous supplementation of melatonin protects mice against Cr(VI)-promoted alterations in testicular histology and germ cell apoptosis, which helps keeping a normal spermatogenesis and male fertility [184]. In agreement with the potential protective effect of melatonin against EDCs effects, Zhang et al. report its protective effect on mouse prepuberal testis in vitro from the negative impact of the plasticizer diethylhexyl phthalate (DEHP) and BPA. Indeed, both EDCs have been found to disturb self-renewal, spermatogonial activity and meiosis, and this is due to, at least in part, the reduced G9a-dependent H3K9 dimethylation [185]. Nevertheless, if melatonin displays any potential therapeutic approach for male infertility in this context, it remains to be fully determined.

DEHP is broadly dispersed in the environment and since 2014 it has been classified as a “substance of very high concern” by the European Chemical Agency [186]. DEHP, a phthalate esters industrial plasticizer, has been strongly correlated to the disruption of rodent and human endocrine systems along with its metabolite mono-(2-Ethylhexyl)-phthalate (MEHP). In animal models and humans, in utero exposure to DEHP has been associated with decreased anogenital distances [187,188] and chryptorchidism [189] and, in rodents, apart from its effects on germ cells, it has also promoted alterations in testicular somatic and spermatogonial stem cells [190]. Nevertheless, some controversial findings have arisen and, apart from the concentrations used/determined and the window of exposure, strain-specific effects may be also responsible for this reality. Indeed, to this extent, the effects of prenatal exposure to DEHP on spermatogenesis and sperm DNA methylation in FVB/N and C57BL/6J mice retrieve different outcomes. In C57BL/6J mice DEHP exposure significantly decreases spermatogenesis, with the number of differentially methylated regions across the genome showing differences towards FVB/N mice. Of note, promoters of vomeronasal and olfactory receptor-coding genes were globally more hypermethylated in the C57BL/6J mice but, conversely, a decrease in the promoters methylation of miRNAs was observed with a more obvious tendency in the FVB/N strain [186].

Finally, in what concerns the impact of naturally occurring EDCs in the epigenetic machinery of male germ cells, the information is scarce. Exposure to zearalenone, a mycotoxin produced by *Fusarium* that acts via the ER signaling pathway to impair mouse spermatogenesis by producing elevated DNA double stranded breaks and decreasing the number of spermatogenic cells [191], has also been shown to promote a global decrease in DNA methylation, an increase in the methylation of histone marker H3K27 and a decrease of estrogen alpha in the testis of pubertal CD1 mice exposed to a dose lower than the no observed effect level (NOAEL). Concomitantly with these findings, the authors report the disruption of meiosis and decreased sperm quality [192]. Moreover, the same group—using the same doses—further explored the consequences of prenatal exposure to zearalenone in ICR mice and found that, irrespective of the strain used, a global reduction in both DNA methylation and estrogen alpha can still be observed, paralleled by elevated histone methylation of H3K9 and H3K27 [193]. Given the effects reported, further attention should be given to zearalenone and caution must be taken when dealing with products contaminated with this EDC.

To our knowledge no consistent studies on phytoestrogens and their influence on epigenetic markers in male germline are reported so far.

### 3.2. Sperm Epigenome is Sensitive to Environmental Exposure and Conveys Environmentally Induced Epimutations Across Generations

Besides the endocrine route, a complex network of cell-to-cell communications regulates germ cell progression and several intra-gonadal signals sustain the production of high quality mature SPZ [194,195,196].

Organic pollutants can bioaccumulate in body fluids due to their lipophilic nature. Important amounts of EDCs have been found in follicular and seminal fluid [197], where SPZ are transported, thus, to constitute an important route of exposure [141].

Several morphological parameters of sperm are affected by EDCs: acrosome integrity, sperm motility correlated with the functional status of mitochondria and sperm fertilizing capability [198], capacitation, lipid peroxidation, chromatin/DNA integrity and chromosomal anomalies. Tavares et al. [141] summarize all these effects, highlighting that mammals are exposed to a multitude of environmental EDCs that might have synergistic deleterious effects on sperm physiology [141]. Interestingly, most effects on sperm quality and fertility are a direct consequence of an intratesticular environment disrupted also in the presence of low doses of BPA [171,172]: testes of rats neonatally exposed to BPA show perturbations in Sertoli cell junctional protein payload, important to create a correct progression of germ cells and, therefore, SPZ of good quality [199,200].

Data demonstrate that toxicants may affect germ cell DNA integrity in the absence of evident alterations on sperm counts, motility, and morphology. Some of the aforementioned effects are, in fact, connected with epigenetic modifications. Studies in both animals and humans suggest that EDCs may interfere with sperm chromatin maturation; rats chronically exposed to vinclozolin or BPA display a disrupted sperm nuclear morphology as a consequence of an impaired chromatin texture [201,202]. A severe sperm chromatin decondensation has also been observed in humans, after environmental exposure to p,p’-DDE [203]. In this regard, an important role may be played by a substantial change in the pattern of HPTMs. Exposure to EDCs has been associated with long-term decreased trimethylation levels of histone H3K27, due to an altered expression in testis of the enhancer of zeste homologue 2 (EZH2) [204]. Depending on BPA exposure duration, change in two different acetylation marks have also been observed: short exposure increases H3K27Ac, whereas long exposure increases H3K9Ac levels [205]. These changes in sperm histone acetylation entail similar changes in early embryos; thus, epigenetic alterations might be paternally transmitted, via SPZ, probably through changes in expression of histone acetyltransferases [205]. Chronic smoking exposure induces in sperm an elevated histone-to-protamine ratio [206], increased global acetylation of H4K8 and H4K12 [207] and abnormal methylation of imprinted genes [208]. As described in testis, Sirt1 seems to mediate most of BPA effects on male reproductive tracts [209].

Interestingly, toxicants also change DNA methylation status in sperm [210], at the level of paternally imprinted genes [211]. Abnormalities in the sperm’s nucleus due to altered DNA methylation are, indeed, one of the primary sources of environmental exposure-dependent male infertility [131]. Human genome includes a high percentage of long-interspersed nucleotide elements (LINE-1) which are heavily methylated in normal conditions; BPA exposure specifically influences LINE-1 methylation in SPZ [212]. DMRs are differentially induced in sperm after toxicant exposure; such an effect may be a direct consequence of an altered expression of DNMTs in testicular germ cells [213]. Therefore, sperm DMRs appear as potential biomarkers to assess the impact of environment on DNA quality and integrity. Exposure of adult mice to particulate air pollution increases global methylation of spermatogonia, which persists through spermatogenesis and remains elevated in mature sperm [214]. Conversely, chromium(III) chloride exposure to adult mice for 2 weeks decreases sperm DNA methylation of the 45 S ribosomal RNA gene [215].

However, the fidelity with which epigenetic states are transmitted is variable; thus, epigenetic signature can undergo alterations known as epimutations. Considering F0, the generation of gestating females, plastic and dioxin exposure causes diseases and abnormalities in male and female animals of F1 and F3 generations [216,217]. It is clear that epigenetic mechanisms may be involved in the transgenerational inheritance of diseases, via SPZ. Exposure of gestating females imposes fetus exposure, as a consequence, during gonadal sex determination; thus, sperm epigenetic programming is altered and transmitted in altered form (epimutation), with an imprinted-like manner, across generations, promoting diseases in adults [216]. In detail, both dioxin and plastics generate 50 and 197 differential DMRs in gene promoters of F3 generation sperm epigenome, respectively [216,217]. Interestingly, sperm DMRs provide potential biomarkers for transgenerational diseases. However, the first evidence for such a mechanism came with the discovery that transient exposure to vinclozolin during embryonic days 8 to 14 (E8-E14), the time of gonadal sex determination in rats, was able not only to induce a reduced fertilizing capability phenotype and testis pathology, but also promote a number of other diseases or tissue anomalies including prostate and kidney diseases, immune system abnormalities, blood anomalies and tumor development up to the F4 generation [218,219]. Since then, apart continuous studies involving vinclozolin exposure [210,220,221], other EDCs, such as 2,3,7,8-tetrachlorodibenzo[p]dioxin (TCDD) [216,222]; PAHs [223,224], glyphosate [225], DDT and p,p’-DDE [225,226,227,228] have also been shown to elicit differential DNA methylation patterns in F3 sperm, and the same has been observed for mixtures containing either the pesticides permethrin and DEET [222,223] or BPA, DEHP and dibutyl phthalate (DBP) [217,222]. Curiously enough, the patterns of methylation exhibit little overlap among exposures, suggesting the lack of specificity of environmentally induced DMRs and a differential DNA methylation fingerprint in SPZ.

Interestingly, DMRs determined at each developmental stage of germ cells isolated from F3 male rats of the DDT lineage showed that most of them are acquired in caudal sperm, suggesting that during epididymal maturation SPZ develop a wide range of epimutations [229,230].

In clear contrast to these findings, no transgenerational effects have been reported after in utero exposure to the anti-androgenic compound flutamide, the fungicide vinclozolin and the pesticides methoxychlor and procymidone, suggesting the existence of a window of sensitivity for anti-androgenic effects [211,231,232,233]. In fact, while some authors report altered DMRs in F1 SPZ and a tendency towards recovery in SPZ from the F2 offspring that persisted through the F3 upon exposure to vinclozolin and methoxychlor, others found no differences in DNA methylation in SPZ of any of the lineages studied [231,232,234].

Developmental exposure to TCDD has been associated to reduced sperm quality/quantity and the occurrence of reproductive defects also in F3 males suggest that sperm conveys the father’s entire contribution to pregnancy, thereby contributing to placental dysfunction in his control partner [235].

Sperm miRNA profile is also influenced by a wide range of environmental challenges such as irradiation (miR-29) [236], exposure to EDCs (miR-29, miR-101) [204,213], stress (miR-375-3p) [237] and exercise (miR-503) [238]. In *Caenorhabditis elegans* germline, piRNAs have also been described as stable key players in the epigenetic memory [239]. Yet, prenatal exposure to vinclozolin induces apoptosis of PGCs along with a reduced fertility rate in adult males of F1 to F3 generations by deregulating specific miRNAs such as miR-23b and miR-21, therefore affecting the Lin28/let-7/Blimp1 pathway [234].

More recently, in utero exposure of rats to vinclozolin has also shown to promote not only differential DNA methylation patterns in sperm but altered ncRNAs in all F1-F3 generations, yet with a clear distinction between the generations suffering from a direct exposure (F1: directly exposed generation fetus; F2: directly exposed germline within the fetus; F3: transgenerational generation sperm). Furthermore, differential histone retention sites have been observed in sperm from F3-vinclozolin offspring but not in the others, showing that alterations may arise later and these are truly transgenerational [240].

Collectively the above results suggest that is not DNA methylation the only epigenetic mechanism involved in this epigenetic transgenerational inheritance phenomenon. Indeed, further supporting this evidence is BPA and its analogs BPE and BPS, which have been implicated in the disruption of DNMTs and alteration of histone marks such as H3K9me2 and H3K9me3 in the neonatal and/or mice adult testis, therefore producing a transgenerational phenotype characterized by an impairment of spermatogenesis and sperm quality [241]. Furthermore, exposure to the herbicide atrazine has been reported to affect both meiosis and spermiogenesis and reduce sperm number in the F3 generation possibly due to a global deregulation of RNA transcription, differential expression of long ncRNAs and global decrease of H3K4me3 in male mice testis [159].

### 3.3. Diet-Induced Epigenetic Modifications Targeting Germ Cells. SPZ as Carriers of Diet-Induced Epimutations Across Generations

Diet-induced epigenetic changes can be mitotically or meiotically heritable. Despite the resetting of cell epigenome between every generation in order to avoid transmission of epimutations, dietary factors can also impact on germ cell epigenetic landscape that is transmitted, in altered form, to the offspring via SPZ [242,243].

In recent years, a wide knowledge has been gained about the impact of diet on health. A correct dietary style, indeed, sustains well-being, energy status, healthy aging and can even be used as a therapeutic approach in diseases [244,245,246,247]. In this context, dietary factors have a specific impact on spermatogenesis in both humans and animal models. Outcomes can vary both in terms of the epigenetic process involved, ranging from direct DNA methylation and histone modifications to ncRNA production—with a direct effect on gene expression—and of cell target specificity, considering that epigenetic landscapes can widely change among different cell types [248].

Fat content in paternal diet provides one of the best examples of the complex network of diet induced epigenetic modulation in spermatogenesis. In 2010 it was demonstrated for the first time that a high-fat diet (HFD) in fathers is able to influence metabolic traits in female offspring. Ng and colleagues demonstrated that fat intake in fathers impacted on glucose-insulin homeostasis in born females, through gene methylation of a regulatory region of the *Il13ra2* gene, directly involved in pancreatic cell remodeling [249]. Although clearly pointing at a non-genetic transmission of epigenetic traits, no specific gametic alteration was highlighted in that first work. Clearer evidence of direct cell-specific, HFD-induced DNA modifications in gametogenesis came from studies showing that HFD induces DNA hypomethylation in testis cells and late spermatids [250,251], as well as a change in miRNA in SPZ [252]. Remarkably, Wei et al. report about a form of HFD-induced prediabetic condition in mice causing altered methylation of several genes including phosphatidylinositol (PI) 3-kinase subunits *Pik3ca* and *Pik3r1* of the insulin pathway, both in SPZ and in the offspring somatic tissues [253].

Paternal diet was also shown to induce specific histone marks variation in mature sperm, especially through H3K9me3 and H3K27me3 [254]. In this regard, a transgenic model of Kdm1A—a histone lysine 4 demethylase—overexpressing the enzyme during spermatogenesis, displayed sperm with an altered H3K4me2 and developmental abnormalities in three subsequent generations [255]. HTPMs can be induced by fat intake, even in the absence of DNA methylation. In particular, specific histone retention has been observed in HFD-modulated spermatogenesis, thus diverging from the required histone-to-protamine transition leading to DNA hyper-compaction in SPZ. H3 retention, as well as differential H3K4me1 enrichment, have been reported in the sperm of HFD-fed male mice at the level of genes involved in embryogenesis regulation [256]. Interestingly, the expression of the HDAC Sirt6 has been shown downregulated in mouse spermatids—transitioning from late round spermatids to early elongating spermatids—upon HFD, thus inducing higher acetylation in immature spermatids [257].

Considering that HFD is a gold-standard experimental model for diet-induced obesity, its application provides a link with disease related phenotypes in male infertility and transgenerational transmission of epigenetic modifications. In this context, besides the effects on semen quality, detectable in terms of DNA fragmentation and enhanced ROS production, obesity displays effects on DNA methylation and histone acetylation [258]. Indeed, acetylation is usually a process required for histone removal in late spermatogenesis and it is linked to higher DNA damage when occurring earlier in time. This sustains that HFD induced epigenetic modifications could even underlie the impaired quality of SPZ from obese men [251].

HFD induced epigenetic transmission entails both chromatin remodeling and ncRNAs within germ cells and SPZ. The contribution of ncRNAs in SPZ seems to be even more direct in transducing the impacts of paternal HFD on the offspring phenotype, in agreement with the evidence that different classes of RNAs are directly involved in DNA, histone methylation and chromatin remodeling [259]. Indeed, several works report a high variety and a differential expression of ncRNAs induced by a HFD in rodent sperm with an upregulation of specific miRNAs in both germ cells and SPZ, thus reprogramming the epigenome of sperm cells and of somatic tissues in the offspring [103,252]. In particular, miRNA let-7c has emerged in mediating such transgenerational epigenetic inheritance induced by paternal HFD [252]. Interestingly, this family of miRNAs has been involved in lipid and glucose metabolism and shows a differential expression in SPZ of HFD fed rats as well as in the SPZ of their offspring. Let-7 has also been implicated in other models of diet-induced transgenerational inheritance, such as in the case of a low-protein diet (LPD) that causes a downregulation of several let-7 miRNAs in SPZ, in association with an altered phenotype of the offspring [260]. Among the ncRNAs isolated from HFD sperm, tsRNAs are also able to induce metabolic disturbance in the offspring, determining a differential expression in genes related to metabolism in early embryos, when injected into zygotes [103].

SPZ from obese men and rats have an altered piRNA signature in comparison to their lean counterparts, suggesting a crucial role for piRNAs, as well, in the epigenetic inheritance of metabolic diseases [252]. A computational analysis revealed that a downstream mRNA target of differentially expressed piRNAs in obese and lean subjects was the Cocaine- and Amphetamine-Regulated Transcript (*Cart*) gene, known as a negative regulator of food intake, involved in obesity [261]. The pattern of expression of piRNAs significantly changes in SPZ from lean and healthy subjects after exercise training [262]. These data clearly add piRNAs to the payload of sperm-borne ncRNAs affected by lifestyle and with a dynamic expression in sperm.

Not only fat but also dietary protein content is able to influence sperm epigenetics, resulting in the regulation of metabolic features of the offspring. Carone and coworkers demonstrated that male mice fed with a low-protein diet generate an offspring with an increased expression of genes involved in lipid and cholesterol synthesis. While this was linked to the level of cytosine methylation in liver DNA, whole-genome characterization of cytosine methylation patterns and RNA content in sperm have also been analyzed but these molecular marks at the level of sperm did not change [254]. Conversely, Radford et al. demonstrate that in utero undernourishment perturbs the adult sperm methylome, with a change in chromatin architecture as a consequence of diet [263]. Accordingly, the periconceptional exposure to famine produces an under-methylation in DMRs of the maternally imprinted *Igf2* gene [264], most of them occurring in regulatory regions of genes with a differential expression during early development. LPD also decreases in sperm the level of H3K27me3 at the promoter of the mitochondrial gene Monoamine oxidase and ELF1 (elongation factor Tu GTP binding domain containing 1) [254]. Low-protein sperm also show an altered content of small RNAs, such as decreased levels of let-7 miRNA and increased levels of 5′ fragments of glycine tRNAs [260].

Interestingly, the suggested mechanism points to epididymosomes as vectors of small RNAs transferred to SPZ during epididymal maturation [260]. More recently, LPD-induced hypomethylation of sperm DNA has been detected in mice, in turn affecting the offspring phenotype by elevated adiposity and metabolic dysfunction [265].

Epigenetic modifications targeting spermatogenesis can even be affected by diet depletion of specific nutrients. Methyl and acetyl groups necessary to fulfil epigenetic dynamics mainly derive from daily consumption of nutrients and micronutrients, comprising methionine, betaine, choline and different types of vitamin B, vitamin D, folate and B12, as well as acetyl-coA [248]. Human sperm quality is improved by a dietary supplementation with folate [266] and vitamin D [267]. In particular, vitamin D has a critical role in the epigenetic routes since genes involved in the vitamin D signaling pathway have several CpG islands in their promoters that can be methylated [268]. Vitamin D epigenetic action has also been linked to histone acetylation [269]. Remarkably, a paternal folate-deficient diet is able to induce a global hypomethylation in liver DNA of rat offspring [270]. Accordingly, in mice such a dietary approach has been shown to induce malformations in the offspring with relevant altered methylation in sperm DNA [271]. This is in line with the evidence of male infertility onset upon mutations in the *Mthfr* gene, encoding the 5, 10-methylenetetrahydrofolate reductase, an important enzyme in folate and methionine metabolism [272]. Nonetheless, the importance of folate is supported by the evidence that its supplementation, together with zinc, determines improved sperm quality in subfertile men [273,274]. Interestingly, paternal LPD perturbs testicular expression of the folate-cycle enzymes *Dhfr*, *Mthfr*, and *Mtr* and of the DNMT1 and DNMT3l [265]. In this context, a growing attention is nowadays focused on the identification of micronutrients or phytochemicals, serving as nutraceutical agents, intended as food-related molecules able to provide medical or health benefits. Polyphenols, such as resveratrol and curcumin, are well known agents able to modulate methyltransferase and histone deacetylase enzyme activities, thus being potential candidates for intentional modulation of epigenetic dynamics in spermatogenesis [248]. Indeed, such molecules are under investigation in the field, showing promising beneficial effects in ameliorating spermatogenesis in obese conditions both in animal models and in humans [275,276,277,278]. Concentration of factors as zinc in seminal fluid modulates protamine formation and dynamically chromatin structure, as a consequence [279]. Accordingly, thiol groups of protamines are engaged into zinc-stabilized bridges, essential to allow high degree of chromatin compaction [280].

Therefore, dietary modulation of epigenetic information in spermatogenesis remains an interesting issue currently under investigation with the aim of better understanding the mechanisms underlying transgenerational transmission of environmental conditioning and of evaluating forms of therapeutic strategies to counteract male sub/infertility.

## 4. Conclusions

Different epigenetic mechanisms—DNA methylation, histone modifications, ncRNAs—are interconnected and form an “epigenetic network” that plays a key role in the proper function of cells, male gamete included.

Epigenetic signature starts to be defined in the testis, during germ cell progression, to reach the highest degree of complexity in SPZ. Interestingly, such a signature is not static, but dynamically changes during SPZ maturation along the epididymis. Here, the contribution of the epithelial epididymal cells is impressive and explicated through epididymosomes.

Several environmental factors—pollutants, stress, diet—affect human health, especially through the epigenetic routes. It is well known, in fact, that epigenome acts as an intermediary between genome and environment and that epigenetic tags can be propagated across generations. A considerable number of reports have discussed the role of the mother’s experience on offspring’s health, but it is only in recent years that studies have addressed the sperm-specific epigenetic signature and how it can be transferred to the oocyte in order to affect embryo development. Future work is still needed to improve our understanding of the underlying molecular mechanisms. In this regard, animal models are a useful instrument to disentangle the skein. To date, many questions are still left unanswered, as why the environmentally induced epigenetic mutations are not corrected during the epigenetic reprogramming occurring between fertilization and implantation, suggesting that much effort is still required to shed light on this topic.

**Figure 3:**
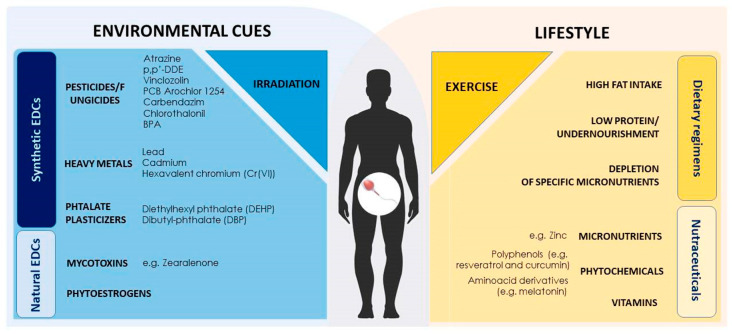
Environmental and dietary factors impacting on spermatogenesis and sperm quality. Several factors are able to influence male fertility through epigenetic modifications targeting different cells involved in spermatogenesis. The figure resumes the relevant environmental agents known to affect male fertility as well as improper dietary regimens. Nutraceutical supplements reported to positively impact on spermatogenesis are also listed.

Furthermore, the environmentally driven epigenetic changes in gametes are not only a fascinating biological topic on its own, but also represent a major global public health problem given their direct impact on disease etiology. Figure 3 summarizes the impact of environment on sperm epigenome and, consequently, on embryo, newborn and adult health through the transgenerational epigenetic inheritance.
